# Abuse, Mental State, and Health Factors Pre and during the COVID-19 Pandemic: A Comparison among Clinically Referred Adolescents in Ontario, Canada

**DOI:** 10.3390/ijerph181910184

**Published:** 2021-09-28

**Authors:** Shannon L. Stewart, Ashley Toohey, Angela Celebre, Jeff W. Poss

**Affiliations:** 1 Faculty of Education, Western University, London, ON N6G 1G7, Canada; sstewa24@uwo.ca (S.L.S.); acelebr@uwo.ca (A.C.); 2 Faculty of Applied Health Sciences, University of Waterloo, Waterloo, ON N2L 3G5, Canada; jwposs@uwaterloo.ca

**Keywords:** health, mental health, COVID-19, interRAI

## Abstract

Throughout the COVID-19 pandemic, population surveys revealed increased levels of anxiety and depression, while findings from large-scale population data analyses have revealed mixed findings with respect to the mental health consequences for children and youth. The purpose of this study was to examine the impact of the COVID-19 pandemic on the well-being and health-compromising behaviors of adolescents (12–18 years) previously referred for mental health services. Data were collected (pre-pandemic *n* = 3712; pandemic *n* = 3197) from mental health agencies across Ontario, Canada using the interRAI Child and Youth Mental Health assessment. Our findings revealed no increased incidence of witnessing domestic violence nor experiencing physical, sexual, or emotional abuse. Further, there were no increases in the risk of self-harm and suicide, anxiety, or depression among our sample of clinically referred youth. Finally, results demonstrated no increase in problematic videogaming/internet use, disordered eating, or alcohol intoxication, and a decrease in cannabis use. Our findings add to the growing body of knowledge as to the impact of the COVID-19 pandemic on children and youth. Further, findings underscore the importance of understanding the nuanced impact of the pandemic on various subgroups of children, youth, and families and highlight the need for continued monitoring of outcomes for these children and youth.

## 1. Introduction

The COVID-19 pandemic continues to have a wide-ranging impact on the well-being of children, youth, and families around the world. When COVID-19 was first declared a pandemic by the World Health Organization (WHO) in March 2020, governments began putting emergency measures in place to stop the spread of the virus [1]. Within the Province of Ontario, this included closures of childcare centers, schools, organized activities, most in-person visits to the doctor or dentist, and non-essential retail stores. Notably, these closures have caused major disruptions to daily routines and widespread social isolation, which can both have a significant impact on mental health [2,3,4]. While a growing body of research shows that the pandemic and associated restrictions have led to increased mental health problems and psychological distress among adults [5,6], studies examining the impact on adolescents are scarce, which is concerning because they represent a particularly vulnerable population [7,8,9].

The period of adolescence has been described as a time of “storm and stress” [10]. Indeed, it is characterized by a number of factors that can place an adolescent at higher risk for developing mental health issues, such as heightened emotional reactivity and lower emotion regulation [11]. Adolescence is also a crucial time for social development, as it is when individuals become more independent from their family, develop relationships with peers based on shared values/ideas, rely more on friends for emotional support, and become more sensitive to peer acceptance and rejection [12,13,14,15]. Taken altogether, the features of this developmental period, coupled with social distancing measures and school closures, potentially place adolescents at greater risk of developing psychological issues during the COVID-19 pandemic.

Recent research has begun to explore the particular challenges that adolescents are facing during this tumultuous time. One study utilized a mixed-methods approach to examine the social-emotional impact of the pandemic from the perspective of adolescents [16]. Findings suggested that the most common challenge among this age group was interrupted in-person interaction. Adolescents reported that connecting with their peers through digital platforms impacted their ability to socialize and obtain emotional connection and support. The second most commonly reported challenge was stay-at-home restrictions, thereby limiting the ability to participate in activities outside of the home with family and friends. Other adolescents found that spending too much time with family was a challenge due to the lack of privacy and personal space. Others reported COVID-related angst (e.g., fear and anxiety with respect to the virus); being “in a funk” (e.g., feeling sad, lethargic, and unmotivated due to the lack of routine); and stress related to school (e.g., emotional and mental difficulties as a result of the transition to virtual classrooms) [16]. Positive aspects and benefits of the pandemic were also highlighted by adolescents as part of this research [16]. For example, some adolescents reported spending more valuable time with family, experiencing more quality time to focus on themselves (e.g., to exercise, or just slow down and relax) and improving friendships (e.g., by the pandemic “testing” and “proving” these relationships) [16].

Although in its infancy, research has also begun to examine the impact of the COVID-19 pandemic on the mental health of adolescents. A systematic review by Jones and colleagues [17] found that adolescents from different countries around the world generally reported heightened stress, anxiety, and depression, as well as increased use of alcohol and cannabis during the pandemic. However, a few studies have found either no difference or an improvement in mental health symptoms during the pandemic. For example, Chen and colleagues [18] did not find a significant correlation between the pandemic and anxiety or depressive symptoms among a sample of adolescents. Another study found that before the pandemic, 38.53% of adolescents reported anxiety symptoms and 51.51% reported depressive symptoms; however, after home confinement, this decreased to 23.73% and 38.29%, respectively [19]. Finally, other research conducted among adolescents in Iceland found a decrease in e-cigarette use, cigarette smoking, and alcohol intoxication during the pandemic, compared to pre-pandemic rates [20].

While the literature shows that the pandemic has largely had a negative impact on the mental health of adolescents within the general population, some studies have found a differential impact among certain sub-groups. Hu and Qian [21] found that the impact of the pandemic on adolescent mental health differed based on the level of mental health problems before the pandemic. More specifically, young persons who had “worse-than-median” mental health before the COVID-19 pandemic experienced a decrease in hyperactivity, conduct problems, peer relationships problems, and emotional problems, but an increase in their prosocial tendency during the pandemic. However, young persons who had “better-than-median” mental health before the pandemic experienced changes in the opposite direction [21]. Another longitudinal study examined the impact of the pandemic among a sample of healthy and at-risk adolescents (i.e., those who experienced early life stress, such as domestic violence, child abuse and neglect, and parental psychopathology) [22]. In contrast to the study’s predictions, healthy adolescents experienced a significant increase in anxiety and depressive symptoms following the onset of the pandemic, whereas these symptoms remained high but stable in adolescents with early life stress. Overall, these findings highlight the fact that the negative impact of the COVID-19 pandemic on adolescent mental health is not universal and that there is likely a myriad of factors that need to be considered in order to understand the nuanced effects on different sub-groups within this vulnerable population.

The aim of the current study was to examine the impact of the COVID-19 pandemic on the mental health and well-being of adolescents previously referred to secondary mental health agencies in Ontario, Canada. A longitudinal design was utilized to compare a vast array of variables pertinent to adolescent mental health prior to and during the pandemic. We also utilized area-based measures of income to examine the relationship between socioeconomic status and pandemic-related changes within this vulnerable population. While the literature has generally shown an increase in mental health symptoms and health-compromising behaviours among adolescents during the pandemic, notable differences have been reported among various sub-groups. Given the inconsistent findings regarding mental health implications for treatment-seeking adolescents, the current study was exploratory in nature.

## 2. Materials and Methods

### 2.1. Sample

Data were obtained from assessments of individuals aged 12 to 18 years accessing mental health services in Ontario, Canada, as part of the standard of care. Individuals were referred to these agencies through a variety of sources, including family and specialty physicians, school staff, other allied health professionals, or parents/primary caregivers. The collected assessment information is used for a variety of purposes by the assessing agencies, including care planning, informing decision making, and tracking individual-level change. The sample included those assessed with the interRAI Child and Youth Mental Health Assessment (ChYMH) [23] and Adolescent Supplement (described below) as part of regular clinical practice in 35 agencies between 17 March 2019 and 16 March 2021. The sample was divided into two time periods, comprising a pandemic year beginning 17 March 2020 (the date Ontario declared a state of emergency as a result of the novel coronavirus), and a pre-pandemic year exactly one year prior. There were 8559 assessments completed for 6133 unique individuals. Individuals could be assessed in both years as well as more than once in a given year. The rationale for using all assessments was that this best represents the flow of cases into these organizations in a consistent way over both years of interest.

Assessors received training on the completion of the ChYMH and Adolescent Supplement. Assessments were completed by a variety of clinicians, including psychologists, nurses, psychiatrists, speech and language therapists, child and youth workers, developmental social service workers, and social workers. All available sources of information were utilized to complete the assessment (i.e., adolescents, family members, community members, document review, and clinical observations).

Secure web-based software was utilized to record assessment information, requiring responses for all essential items before the record could be authorized as complete. Before making the data available for analysis, personal identifiers were removed. Western University’s ethics board granted approval for the secondary analysis of data collected in various agencies throughout the Province of Ontario (REB #106415).

### 2.2. Measures

The interRAI ChYMH [23] is a needs-based assessment comprised of over 400 clinician-rated items addressing a number of domains related to child and youth mental health (e.g., psychiatry, social, familial relations, environmental, academic, medical). Information from this assessment system can be used for individualized client assessment, outcome measurement, quality indicators, and resource allocation. A number of algorithms and scales are embedded in the ChYMH to indicate the level of risk based on symptom frequency and severity. These scales and algorithms assist with goal setting for intervention and treatment planning. The interRAI Adolescent Supplement is integrated into the ChYMH and completed for all youth who are 12 years of age or over, or those under 11 years of age if they are engaging in more mature behaviors (e.g., illicit drug use).

Polyvictimization was classified as having experienced two or more of four types of personal trauma: witnessed domestic violence, sexual assault/abuse, physical assault/abuse, or emotional abuse. It was assigned for two look-back periods: lifetime, and in the last year.

Five self-report items were available, recording the client’s personal assessment, including an option where the client would not or could not respond. These included three mood items (little interest/pleasure in things normally enjoyed, anxious/restless/uneasy, and sad/depressed/hopeless), a rating of sleep quality, and an overall rating of health.

Neighborhood median income was obtained by linking the first three digits of the client’s home postal code to public-use 2016 Canadian census tables [24]. Clients were subsequently assigned to quartiles based on the distribution of these data.

Scales and Algorithms: All calculated scales are defined such that higher scores denote more acute levels. For ease of reporting, the scales were dichotomized at a practical point and the proportion in the high range reported.

The Risk of Suicide and Self-Harm in Kids (RiSsK) [25] is a scale for measuring risk of self-harm. It uses multiple items from the assessment, including an attempt to kill themself, self-harm without an attempt to kill themself, consideration of self-injury, others concerned about self-injury, family overwhelmed, and any self-injurious behaviors.

The Risk of Injury to Others (RIO) [26] algorithm is an empirically supported decision-making tool that measures the risk of harm to others in clinically-referred children and youth populations. The algorithm uses measures of violent ideation, threatened violence, violence to others, verbal abuse, socially inappropriate or disruptive behavior, family overwhelmed, impulsivity, and physical abuse.

The frequency and severity of aggressive and disruptive behavior were assessed using the Disruptive/Aggressive Behaviour Scale (DABS) [27]. Items include physical abuse, verbal abuse, socially inappropriate or disruptive behavior, destructive behavior toward property, and outbursts of anger.

The Depression Severity Index (DSI) [28] measures depressive symptoms in child populations, including sad or pained facial expressions, making negative statements, self-deprecation, guilt/shame, and hopelessness.

The Anxiety Scale [29] measures anxiety through six items: anxious complaints or concerns, unrealistic fears, obsessive thoughts, intrusive thoughts or flashbacks, episodes of panic, and nightmares.

Hyperactivity and distractibility are assessed by the empirically supported Hyperactive/Distraction Scale (HDS) [27]. Items include impulsivity, ease of distraction, hyperactivity, and disorganization.

### 2.3. Analysis

Measures of interest were compiled for the pandemic year and for the year prior. Given there were fewer assessments in the pandemic year, and because this decline differed, notably by sex, a corrective weight was applied to the pandemic year to adjust for the likelihood of selection. We chose to combine age, sex, and area income quartile and use this as a classification from which weights were assigned to cases in the pandemic year equal to the inverse of their likelihood of selection compared to the year prior. Given that this would not normally be expected in a year-over-year sample, it is likely that the pandemic conditions themselves influenced the likelihood of client selection. As such, the demographic and area income variables that were available were used to correct this. We examined both the unweighted and the weighted samples for differences between the two years, using chi-square tests for dichotomous and categorical variables and t-test for the continuous variable of age. 

## 3. Results

Comparing pandemic and pre-pandemic years, no significant difference was observed in the proportion of individuals contributing 1, 2, 3, or 4 (the maximum) assessments per year (chi-sq = 4.12, *df* = 3, *p* = 0.249). Table 1 presents the sample demographics. Our sample consisted of 3712 individuals pre-pandemic and 3197 individuals during the pandemic. However, our analyses were based on a sample of 4612 assessments in the year pre-pandemic and 3947 assessments during the pandemic year. There was a trend toward a significant difference in mean age, as evaluated with the Student *t*-test. However, this difference is not clinically significant. There was, however, a notable decline in the proportion of males assessed. There was also a trend toward a significant difference in representation from the lowest income neighborhoods during the pandemic as compared to pre-pandemic data. Categorical differences were evaluated using chi-squared tests.

Table 2 provides proportions of a variety of characteristics in the pre-pandemic and pandemic year, as well as a proportion in the pandemic year that reflects an adjustment for age, sex, and neighborhood income. All tests of significance use chi-square tests, with adjusted p-values adding calculated weights based on the inverse likelihood of selection (age/sex/income quintile) in the pandemic period. Measures showed a significant decline (at a *p* ≤ 0.001 level), after adjustment, in the pandemic year with respect to a variety of issues including the following: recent child protection involvement, caregiver distress, family members overwhelmed, youth criminal justice referrals, bullying and victimization, conflicts with family, parents, or peers, involvement with the community, and drugs and alcohol. Scale measurements showed assessed adolescents did not demonstrate an increased risk in the pandemic year, after adjustment, for harm to self or others, anxiety, or prevalence of depressive symptoms. Supportive relationships with peers showed higher prevalence, after adjustment, in the pandemic year.

Table 3 presents additional measures specific to schools. All tests of significance use chi-square tests, with adjusted p-values using calculated weights based on the inverse likelihood of selection (age/sex/income quintile) in the pandemic period. These items consistently showed notably lower proportions in the pandemic year, compared to the pre-pandemic period with respect to the following school-related issues: absenteeism, poor school productivity, intention to quit, conflict with staff, refusal to attend, being removed due to behavior and feelings of strong dissatisfaction with schools. Self-reported items that show fair or poor overall health and fair or poor sleep are also summarized in Table 3.

## 4. Discussion

The purpose of the current study was to add to a growing body of literature examining the impacts of the COVID-19 pandemic on adolescents’ health/well-being and health-compromising behaviors. Comparisons of pre-pandemic and pandemic data revealed no increase in the incidence of witnessing domestic violence, nor experiencing physical, sexual, or emotional abuse. Further, results presented herein demonstrate reduced interpersonal conflict as well as a decline in experiences of bullying and being bullied. Moreover, among our sample of referred adolescents for mental health services, our results demonstrated an increase in supportive relationships with peers. While several studies demonstrated an increased risk of family violence following the start of the COVID-19 pandemic, this was not present among our sample of adolescents, a finding that is consistent with that of Cohen and colleagues [22]. With respect to mental state indicators, our results demonstrated no increase in the risk of harm to self or others, anxiety, or depressive symptoms. These findings are consistent with previous findings demonstrating that those adolescents with poorer than average mental health prior to the COVID-19 pandemic experienced a decrease in a variety of mental health indicators and an increase in prosocial tendency during the COVID-19 pandemic [21]. It is also possible that participants of the current study have previously received one or more forms of mental health support (e.g., psychotherapy, psychopharmacological) and thus already have needed services resulting in improved coping to manage changes brought on by the COVID-19 pandemic. Consequently, few changes in mental state indicators were found.

Findings presented herein found no increase in disordered eating habits, problematic video gaming and internet use, or alcohol intoxication, with a noted decrease in cannabis use. Further, participants’ own ratings of their health and sleep were not as poor during the pandemic year compared to their pre-pandemic status. Previous research findings have demonstrated that good sleep and positive interactions with peers were both related to a lack of increase in anxiety symptoms [22]. Further, previous COVID-19 pandemic findings have demonstrated a link between the use of video games as a coping strategy and increases in depression and anxiety [22]. Among our sample, we saw no change in problematic video gaming and a reduction in depressive symptoms. Further, findings from the current study demonstrated a decrease in self-reported poor sleep, an increase in supportive relationships with peers, and a decrease in self-reported feelings of anxiousness. Thorisdottir and colleagues [20] also found a decrease in substance use during the pandemic. These authors suggested that a potential unintended benefit of the pandemic might be that factors associated with social distancing, lockdowns, and reduced peer contact may act as a protective factor against engaging in substance use with peers.

With respect to school-specific indices, our findings indicated that during the pandemic, adolescents referred for mental health supports were less likely to be absent or refuse to attend school, less frequently reported intent to quit school, were removed less frequently from school due to problematic behavior, engaged in conflict with staff less frequently, and had reduced poor work productivity. Given that students engaged in virtual learning for much, or all, of the pandemic year, some of these findings are not surprising. Perhaps the most noteworthy findings are related to the decrease in reported intention to quit school and decreased poor work productivity. It is possible that by not engaging in in-person learning, a variety of additional stressors and barriers to work completion were removed. Conversely, it is also possible that the demands were not as stringent within the context of online learning, resulting in a more lenient, tolerant approach when experiencing online or virtual classrooms, as well as minimal interaction and school engagement.

Of note, emerging research outlining the mental health impacts of the COVID-19 pandemic on children and adolescents has varied, with some findings indicating significant negative impacts, and others finding little or no negative impact of the pandemic. These mixed results suggest that children and adolescents at different mental health stages may experience the pandemic differently and thus have different mental health needs [30]. It is also possible that given the higher prevalence of mental health issues in adolescence, as compared to childhood, there may be more room for improvement among adolescent samples than among samples of children [31], particularly in those accessing mental health services. Furthermore, emerging research findings have underlined that certain sub-populations, including black children [32], children with developmental disabilities, as well as those from ethnically diverse backgrounds [33], are at heightened risk for negative mental health sequelae during the pandemic. Taken together, these findings underscore the importance of continued monitoring of the mental health needs of vulnerable children and youth.

While the data used in this study came from a large sample and was collected using a comprehensive and robust assessment tool, this study is not without limitations and thus raises several important questions for future research in this area. First, our data represents the entire first year of the pandemic, which spanned across two academic years. Further, the data presented herein covered a time period in which stay-at-home orders varied. Hence, it is possible that a more detailed breakdown of time periods may reveal differences in factors such as interpersonal conflict, health-compromising behaviors, and mental state indicators. Additionally, the data within this study incorporates only the first year of the pandemic, and as such, impacts of the COVID-19 pandemic beyond the first twelve months are not accounted for. New, innovative research will be needed, likely for years to come, in order to gain a more comprehensive understanding of the impacts of the COVID-19 pandemic on adolescents. Additionally, our sample consisted of adolescents referred for mental health services in Ontario, Canada, and consequently, results may not be generalizable to other populations of adolescents. Finally, potential differential impacts of the pandemic for individuals of various ethnicity were not examined, which is a limitation.

## 5. Conclusions

Findings from the current study demonstrate that for clinically-referred adolescents, restrictive COVID-19 pandemic measures (i.e., virtual learning, home confinement) may act as a protective factor against worsening mental state indicators, health-compromising behaviors, interpersonal conflict, and negative school indicators during the adolescent years. This has significant implications for care planning for youth seeking mental health treatment. Taken together, our findings underscore the importance of understanding the nuanced impact of the pandemic on various subgroups of children, youth, and families. Given the COVID-19 pandemic is still ongoing, these findings highlight the need for continued monitoring of outcomes for these children and youth longitudinally to determine the long-term impacts of the pandemic on the mental health needs of vulnerable youth, especially as they return to in-person learning.

## Figures and Tables

**Table 1:** Sample demographic description, by year.

Demographics	Pre-Pandemic (Mar 17/19–Mar 16/20)	Pandemic (Mar 17/20–May 16/21)	*p*
Mean age (std)	15.2 (1.69)	15.1 (1.73)	0.027
12 to 13 year olds	29.4%	31.6%	0.080
14 to 15 year olds	36.7%	35.2%	
16 to 18 year olds	34.0%	33.2%	
Male	44.7%	38.5%	**<0.0001**
Female	54.9%	61.1%	
Other	0.4%	0.4%	
Low Neighbourhood Income Quartile	26.4%	23.5%	0.010
2nd Neighbourhood Income Quartile	25.6%	25.7%	
3rd Neighbourhood Income Quartile	23.6%	25.6%	
High Neighbourhood Income Quartile	24.5%	25.2%	

Note. Bolded *p* values denote a statistically significant difference between pre-pandemic and pandemic data at a *p* ≤ 0.001 level.

**Table 2:** Selected descriptive characteristics, by year, with and without adjustment.

Descriptives	Pre-Pandemic (Mar 12/19– Mar 16/20)	Pandemic (Mar 17/20– May 16/21)	*p*	Pandemic Adjusted (Mar 17/20– Mar 16/21)	
*n*	4612	3947		3947	
Legal guardian mother or father only	31.9%	29.5%	0.018	30.0%	0.048
Current custody dispute for this child/youth	3.1%	3.1%	0.972	3.0%	0.893
Current guardian is child protection	3.8%	3.2%	0.094	3.4%	0.256
Child protection involvement last 90 days	18.4%	15.7%	**0.001**	15.7%	**0.001**
Caregiver expresses feelings of distress	33.1%	26.8%	**<0.0001**	26.9%	**<0.0001**
Family members report feeling overwhelmed	36.3%	30.3%	**<0.0001**	30.7%	**<0.0001**
Major life stressor last 90 days, parent/prim cgvr	27.1%	27.7%	0.551	28.1%	0.307
Parental addiction in the last 30 days	4.7%	4.0%	0.103	4.0%	0.086
Economic trade-offs last 30 days	3.3%	2.6%	0.083	2.7%	0.095
Referral reason: Youth criminal justice	8.7%	6.2%	**<0.0001**	7.2%	0.007
Victim of bullying in the last month	7.6%	3.9%	**<0.0001**	3.9%	**<0.0001**
Bullying of peers in the last month	5.3%	3.0%	**<0.0001**	3.2%	**<0.0001**
Conflict or criticism with family last 3 days	36.0%	33.0%	0.005	33.0%	0.003
Conflict between parents last 3 days	21.6%	19.4%	0.015	19.2%	0.006
Conflict with peers last 3 days	16.8%	12.2%	**<0.0001**	12.4%	**<0.0001**
Supportive relationships with peers last 3 days	70.0%	74.6%	**<0.0001**	73.9%	**<0.0001**
Has at least one regular friend last 3 days	78.3%	81.1%	**0.001**	80.4%	0.012
Social inclusion by peers last 3 days	73.3%	76.6%	**0.000**	76.2%	**0.001**
Reports strong sense of involvement in community	35.2%	32.8%	0.036	32.4%	0.010
Witnessed domestic violence in the last year	4.4%	3.6%	0.058	3.6%	0.060
Sexual assault/abuse in the last year	3.9%	3.9%	0.861	3.5%	0.297
Physical assault/abuse in the last year	6.5%	4.9%	0.002	5.0%	0.003
Emotional abuse in the last year	13.1%	11.7%	0.055	11.7%	0.045
Polyvictimization (2 or more of the four above) in the last year	7.1%	5.7%	0.011	5.6%	0.006
Polyvictimization (2 or more of the four above) in their lifetime	30.2%	28.8%	0.151	28.7%	0.123
Binge eating last 30 days	5.5%	5.4%	0.891	5.1%	0.432
Distorted body image last 30 days	7.7%	9.4%	0.004	8.8%	0.053
Fasting/major restrictions of diet last 30 days	5.3%	6.3%	0.055	6.0%	0.176
Sexual activity last 90 days: promiscuity	3.1%	2.2%	0.009	2.2%	0.010
Sexual activity last 90 days: acts for money	0.5%	0.3%	0.121	0.3%	0.100
History of sexual violence/assault as perpetrator	2.3%	1.9%	0.260	2.3%	0.937
Inhalants, hallucinogens, crack, stimulants, or opiates in the last 30 days	2.2%	1.1%	**0.0001**	1.3%	**0.0007**
Cannabis in the last 30 days	18.7%	14.0%	**<0.0001**	14.9%	**<0.0001**
Alcohol to intoxication in the last 30 days	8.1%	5.5%	**<0.0001**	5.8%	**<0.0001**
Caffeine or energy drinks in the last 3 days-None	70.6%	73.1%	0.061	72.8%	0.136
1 or 2	22.7%	21.3%		21.3%	
3 to 5	5.4%	4.7%		4.8%	
6 or more	1.3%	1.0%		1.2%	
Problem video gaming or internet use-none	69.8%	69.9%	0.955	68.0%	0.297
Minimal	17.8%	18.0%		18.9%	
Moderate	9.2%	9.2%		9.8%	
Severe	3.2%	3.0%		3.3%	
Risk for Self Harm Scale (RISSK) 1+	65.6%	66.1%	0.619	65.0%	0.570
Risk for injury to others (RIO) 1+	40.9%	33.9%	**<0.0001**	35.4%	**<0.0001**
Destructive/Aggressive Scale (DABS) 4+	32.2%	26.4%	**<0.0001**	27.3%	**<0.0001**
Depressive Symptom Inventory (DSI) 4+	50.4%	49.1%	0.202	48.1%	0.024
Anxiety Scale 3+	57.3%	58.5%	0.284	57.8%	0.667
Hyperactivity/Destructive Scale (HDS) 9+	21.0%	21.3%	0.743	21.9%	0.283

Note. Bolded *p* values denote a statistically significant difference between pre-pandemic and pandemic data at a *p* ≤ 0.001 level.

**Table 3:** Selected school and self-reported items, by year, with and without adjustment.

	Pre-Pandemic (Mar 12/19– Mar 16/20)	Pandemic (Mar 17/20– May 16/21)	*p*	Pandemic Adjusted (Mar 17/20– Mar 16/21)	*p*
*n*	4612	3947		3947	
Increase in school lateness/absenteeism	30.2%	19.7%	**<0.0001**	20.2%	**<0.0001**
Poor school productivity	29.9%	25.3%	**<0.0001**	26.1%	**<0.0001**
Intent to quit school	9.1%	4.7%	**<0.0001**	4.9%	**<0.0001**
Conflict with school staff	11.7%	5.4%	**<0.0001**	5.7%	**<0.0001**
Child/youth refused to attend school	12.5%	8.4%	**<0.0001**	8.9%	**<0.0001**
Child/youth removed from school due to behavior	3.2%	1.8%	**<0.0001**	1.9%	**<0.0001**
Strong dissatisfaction with school-no	75.0%	81.1%	**<0.0001**	80.6%	**<0.0001**
Child/youth only	19.6%	15.8%		16.1%	
Parent/primary caregiver only	1.1%	0.7%		0.7%	
Both child/youth and parent/primary caregiver	4.3%	2.5%		2.6%	
Self-reported:					
Little interest in things-not in last 3 days	57.9%	56.8%	0.347	56.7%	0.178
Not in last 3 days, but often feels that way	11.4%	10.5%		10.4%	
In 1–2 of last 3 days	11.2%	11.9%		11.7%	
Daily in the last 3 days	8.7%	9.4%		9.2%	
Could not/would not respond	10.8%	11.4%		12.0%	
Anxious/restless/uneasy-not in last 3 days	29.8%	31.5%	0.125	32.1%	0.005
Not in last 3 days, but often feels that way	14.9%	15.1%		15.1%	
In 1–2 of last 3 days	21.8%	21.5%		21.0%	
Daily in the last 3 days	22.8%	20.7%		20.1%	
Could not/would not respond	10.6%	11.2%		11.7%	
Sad/depressed/hopeless-not in last 3 days	43.0%	41.8%	0.098	42.2%	0.055
Not in last 3 days, but often feels that way	14.6%	13.4%		13.2%	
In 1–2 of last 3 days	16.9%	18.9%		18.5%	
Daily in the last 3 days	14.8%	14.8%		14.4%	
Could not/would not respond	10.8%	11.2%		11.8%	
Health—excellent	8.7%	8.4%	0.004	8.7%	**<0.0001**
Health—Good	56.7%	58.3%		58.2%	
Health—Fair	20.3%	18.2%		17.5%	
Health—Poor	4.0%	3.1%		3.0%	
Could not/would not respond	10.4%	12.1%		12.5%	
Sleep—excellent	5.4%	5.9%	0.013	6.1%	**0.001**
Sleep—Good	38.9%	40.0%		40.0%	
Sleep—Fair	27.3%	25.4%		24.9%	
Sleep—Poor	18.5%	17.0%		16.9%	
Could not/would not respond	10.0%	11.6%		12.1%	

Note. Bolded *p* values denote a statistically significant difference between pre-pandemic and pandemic data at a *p* ≤ 0.001 level.

## Data Availability

Due to the highly sensitive and confidential nature of the data, as well as the ethical requirements required for use, data will not be made freely available. Moreover, participating mental health agencies required that data not be made freely accessible.
